# The Role of Anchor-Tipped Larval Hairs in the Organization of Ant Colonies

**DOI:** 10.1371/journal.pone.0041595

**Published:** 2012-07-25

**Authors:** Clint A. Penick, R. Neale Copple, Raymond A. Mendez, Adrian A. Smith

**Affiliations:** 1 School of Life Sciences, Arizona State University, Tempe, Arizona, United States of America; 2 Work as Play, Portal, Arizona, United States of America; Université Paris 13, France

## Abstract

The spatial organization within a social insect colony is a key component of colony life. It influences individual interaction rates, resource distribution, and division of labor within the nest. Yet studies of social insect behavior are most often carried out in artificial constructions, which may change worker behavior and colony organization. We observed how workers of the ant *Pheidole rhea* organized brood in nests with deep chambers and textured walls that were designed to mimic their natural constructions more closely. Instead of clumping larvae into piles on the chamber floor, workers suspended fourth-instar larvae from the vertical walls and ceiling of each chamber while young larvae and pupae were clumped at the base. Fourth-instar larvae possess five rows of anchor-tipped hairs on their dorsal side, and we predicted that these hairs functioned to attach larvae to the nest walls. We gave larvae “haircuts,” where only the anchor-tipped hairs were removed, and then tested their ability to adhere to a textured surface raised to an angle of 90° and then 120° with respect to the horizontal plane. Larvae whose hairs had been clipped came unattached in almost all trials, while larvae whose hairs remained intact stayed attached. This confirmed that anchor-tipped hairs functioned to attach larvae to the walls of the nest. The presence of anchor-tipped hairs is widespread and has been documented in at least 22 genera from the ant subfamily Myrmicinae, including species that occur in a variety of environments and represent a broad range of nesting habits. Based on our results, it is likely that many species exhibit this larval hanging behavior, and this could impact colony characteristics such as spatial organization and the care of developing larvae by nurse workers.

## Introduction

Collective behavior in social insect societies results from interactions among group members as well as their interaction with the nest environment [1]. In addition to nest architecture, which defines the physical space a colony inhabits, the arrangement of individuals and items within the nest may also influence colony-level traits [2]. Larvae represent the primary growth stage during development, and their location in the nest may be important for optimizing individual growth rate and ultimately contributing to colony growth and fecundity. The organization of larvae within the nest, therefore, is thought to have functional significance and could influence colony-level traits.

The structure and organization of conspicuous, aboveground nests built by wasps and bees have been well described, and the contents of these nests are arranged in well-defined patterns [3], [4], [5]. The comb nests of the honey bee *Apis mellifera*, are organized with brood located at the center of the comb, nectar stores at the periphery, and pollen stores are located between these two sections [3], [6]. This arrangement facilitates proper thermoregulation of the brood and minimizes the distance between larvae and their primary protein source: pollen [7]. Adult workers may assess the pollen needs of the colony based on the ratio of pollen stores to the number of larvae present. The spatial arrangement of honey bee nests, with pollen stores located at the periphery of brood cells, may facilitate comparisons between these factors to mediate foraging decisions (reviewed in [7]).

In comparison to studies on wasps and bees, observations of ant nests in the field have proven more difficult because their nests are generally constructed underground and out of view. As a result, most studies of in-nest ant behavior have been conducted in artificial laboratory nests. Observations in these nests have shown that ant workers are able to discriminate among brood and group them based on life stage [8]. For example, workers of the ant *Temnothorax* organize their brood into a pile of concentric rings: eggs and small larvae are at the center, pupae and pre-pupae are grouped in the middle ring, and medium-sized larvae form the outermost ring [9], [10]. Because medium sized larvae represent the primary growth stage of *Temnothorax*, their location at the periphery of the brood pile may place them in greater contact with workers that bring food [9].

While laboratory studies like the one described above are generally performed in two-dimensional nest constructions, the majority of ant species inhabit nests with three-dimensional properties including variation in chamber depth and size [11]. Using artificial nests may have unintentional consequences for the expression of natural behaviors. In order to address this issue, we observed colonies of the ant *Pheidole rhea* in nests designed to mimic the nests they construct in nature. These nests featured deep chambers and textured walls, which approximates their natural constructions more closely (personal observation). In addition to observing larval organization inside the nest, we also determined the number of larval instars in *P. rhea* and investigated the function of specialized, anchor-tipped larval hairs. Wheeler and Wheeler [12] suggested that anchor-tipped larval hairs might enable workers to hang larvae on the walls of their nests. Diverse hair morphologies have been documented in numerous ant species, but in most cases structure has yet to be experimentally linked with function. Anchor-tipped hairs are widespread within the ant subfamily Myrmicinae, and this study is the first to address the function of these hairs in their nest environment. We document the morphology and presence of these hairs with respect to larval instar and caste, and we discuss the significance of anchor-tipped larval hairs with respect to their widespread presence in other ant genera.

## Materials and Methods

### Rearing Conditions

Two mature colonies of *P. rhea* were maintained in the laboratory at 24°C. Incipient colonies (founding queen plus brood) were originally collected in Rio Rico, AZ in July 2005 (31° 27′ 38″ N, 110° 59′ 51″ W, elevation 1064m) and were reared as described by Morgan [13]. Mature colonies were housed in dental-plaster nests with molded chambers that were covered by glass and darkened by red-acetate. Nests were attached to large foraging arenas (35×200 cm), which were kept in constant light. Water, sugar-water, mixed birdseed, Bhatkar diet [14], and pieces of crickets (*Acheta domesticus*) and beetle larvae (*Zophobas morio*) were provided *ad libitum* to colonies in the foraging arena.

### Larval Morphology and Instars

Larvae were first examined using a light microscope (Leica MZ9.5, 60× magnification) to determine basic external morphology. Observations were carried out in finer detail using a scanning electron microscope (SEM). Larval specimens were sputter-coated with gold dust and imaged using a Phillips XL30 ESEM-FEG.

In order to determine larval instars, larvae of all size classes were examined under a light microscope to detect differences in morphology. Photographs were taken of larvae against a grid background for scaling, and measurements of maximum head width and body length were determined using ImageJ software (version 1.44, 2010). Body length was measured by a straight line drawn from the maximum anterior edge to the maximum posterior edge of a larva in ventral view. A frequency histogram of larval head width measurements was used to distinguish peaks that may identify separate instars in cases where there were no obvious differences in morphology.

### Larval Hanging and the Function of Anchor-tipped Hairs

Two colonies were placed into nests that featured irregularly shaped cylindrical chambers 4–5 cm in depth with a diameter of 6 cm. The inside of each chamber was coated with sand mixed with plaster to mimic the texture of natural nests. Colonies were observed through a glass plate covering the top of the chamber to determine how larvae were organized inside the nest.

In order to test the function of anchor-tipped larval hairs we performed a manipulation experiment where larvae received “haircuts.” We removed all anchor-tipped hairs using micro surgical scissors without removing other hairs (such as bifid hairs). Control larvae that did not receive haircuts were removed from the nest for the same period of time as clipped larvae. Larval hanging ability was tested by placing larvae with their dorsal side against a textured surface (created by coating one side of packaging tape with natural sand, which performed better than commercial sandpaper). The textured surface could be raised using a hinge so the hanging ability of larvae could be tested at different angles with respect to the horizontal plane. Two control larvae and two “haircut” larvae were tested during each trial. The trials were conducted blind so that the experimenter did not know which larvae had their hairs removed. After larvae were placed onto the textured surface, the surface was raised to 90° with respect to the level plane; after holding for 10 seconds, the angle was increased to 120° and held for an additional 10 seconds. We then recorded whether a larva stayed attached to the surface or fell. For each set of larvae we repeated the test for three separate trials and recorded the proportion of trials in which they stayed attached. A total of 20 larvae were tested for each treatment group.

## Results

### Larval Morphology and Instars

**Figure 1:**
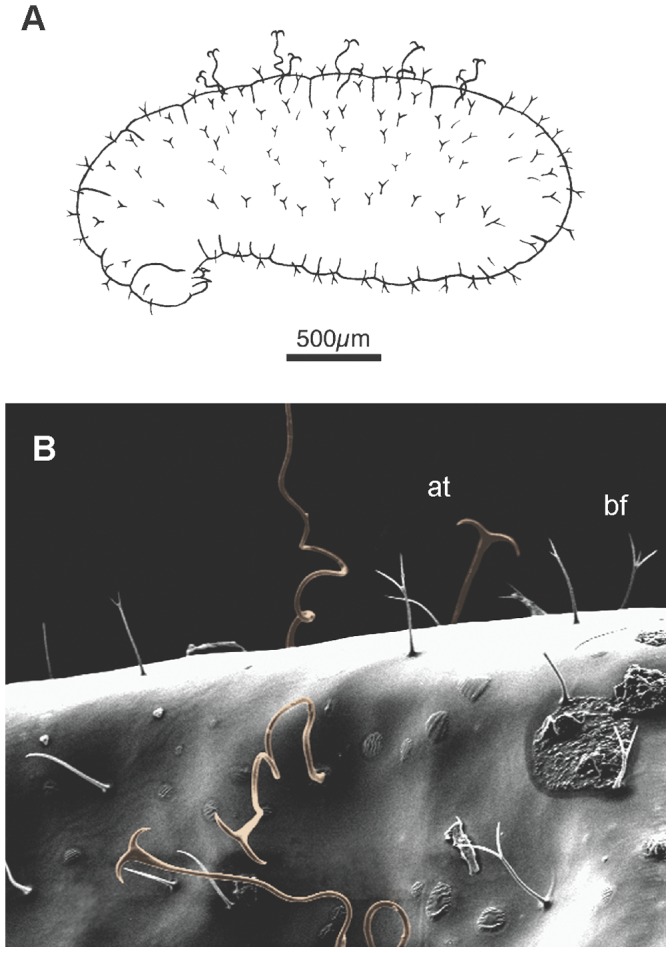
Morphology of fourth-instar minor worker larvae. A) Line drawing illustrating the distribution of larval hairs (note that anchor-tipped hairs occur on the dorsal region of the larva and are distributed in five rows on abdominal segments I-V). B) SEM image showing detail of larval hairs with anchor-tipped (at) and bifid (bf) hairs indicated. Anchor-tipped hairs are color-highlighted.

The final-instar larvae of *P. rhea* are pheidoloid in shape (larval morphological terms based on [12]); the head is located ventrally at the end of a thick neck. The body was covered with bifid hairs with few simple hairs interspersed. Final-instar larvae possessed a transverse row of four anchor-tipped hairs on the dorsal surface of abdominal segments I-V (Fig. 1). Anchor-tipped hairs had tortuous shafts and were approximately four times the length of other larval hairs. Early-instar larvae lacked anchor-tipped hairs and were covered by sparse, simple hairs.

**Figure 2:**
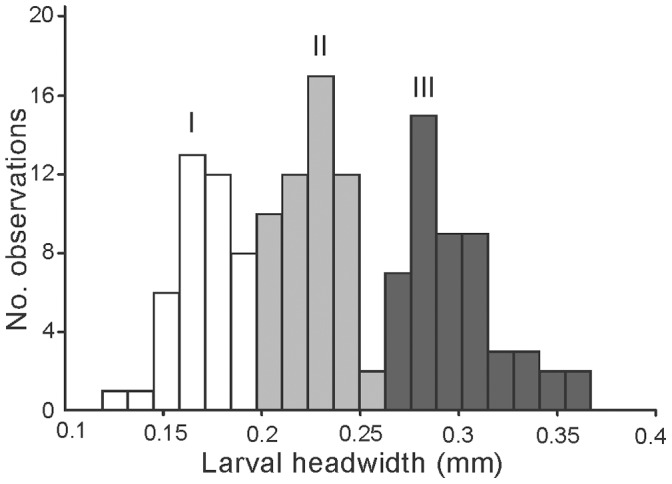
Frequency distribution of early-instar larval head widths. Three peaks were identified in the distribution of larval head widths to indicate the groupings we used to delineate early instars (roman numerals indicate instars I-III). Fourth-instar larvae were clearly differentiated from early instars by the presence of anchor-tipped hairs and were not included in this figure.

The presence of anchor-tipped hairs clearly distinguished final-instar larvae from early instars. A frequency histogram of head width measurements from final-instar larvae revealed a normal distribution with a single peak, which provides further support that these larvae represent a single instar. When head width measurements (Table 1) for early-instar larvae (i.e. excluding larvae that had anchor-tipped hairs) were plotted in a frequency histogram, it revealed a distribution with three overlapping peaks, which suggests the presence of three early instars (Fig. 2), for a total of four larval instars in this species.

**Table 1:** Larval morphometrics.

Instar	Head width (mm)		Length (mm)		*N*
	mean±SD	range	mean±SD	range	
**I**	0.17±0.02	0.12–0.20	0.47±0.07	0.33–0.63	41
**II**	0.23±0.02	0.20–0.27	0.75±0.17	0.48–1.31	57
**III**	0.30±0.02	0.27–0.37	1.09±0.19	0.63–1.41	47
**IV**	0.41±0.04	0.33–0.57	2.11±0.41	0.99–3.12	96
**IV (soldier)**	0.38±0.02	0.35–0.43	5.03±0.54	4.42–6.03	10
**Sexual (male)**	0.32±0.03	0.28–0.39	5.84±0.45	5.01–6.45	12

Examination of final-instar soldier larvae revealed that they also possess anchor-tipped hairs (five rows of four hairs, identical to minor worker larvae), and larval head width measurements fit within the distribution of minor fourth-instar larvae (Table 1). This indicates that soldier larvae most likely represent enlarged fourth-instar larvae, and they are not a supernumerary instar (supported by [15]). We did not distinguish between soldier and supersoldier larvae for this study, though both forms occur in this species [16].

One colony produced male (sexual) larvae in the laboratory in the summer of 2011. Male larvae were slightly larger than fourth-instar soldier larvae based on total length, though their range in length overlapped, and their head widths ranged between third- and fourth-instar head widths (Table 1); however, male larvae lacked anchor-tipped hairs. Sex was determined at the pupal stage, when male sexuals could be distinguished from female sexuals. Only males were produced, and therefore we did not investigate the larval morphology of female sexuals for this study.

### Hanging Behavior

When colonies were kept in nests with a flat chamber (∼1 cm in depth), workers clumped larvae and pupae into piles on the nest floor. However, when colonies were given deep nest chambers (4–5 cm walls) coated with sand, workers of *P. rhea* hung larvae from the inner walls of the nest with their dorsal side attached to the substrate. These larvae were almost exclusively fourth-instar, though early-instar larvae were sometimes observed on the walls. In many cases, larvae could be seen with food resting on their ventral side, and they were observed feeding while suspended to the walls. Soldier larvae and male larvae were never observed attached to the nest walls.

### Function of Anchor-tipped Hairs and their Occurrence Across Genera

**Figure 3:**
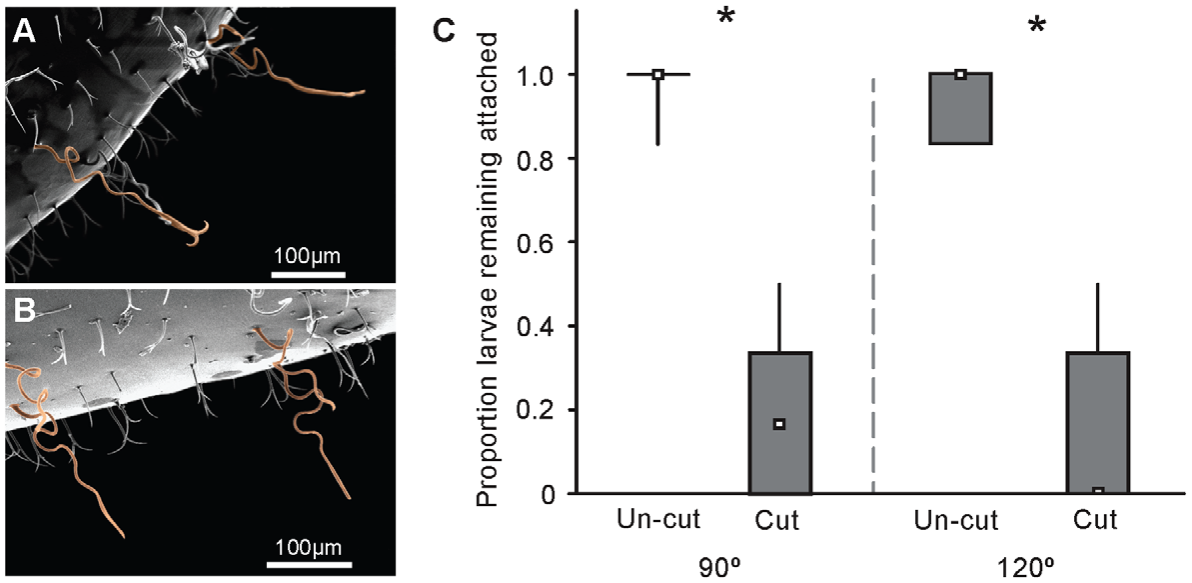
Function of anchor-tipped larval hairs. A) SEM image showing a larva with anchor-tipped hairs intact (color-highlighted). B) SEM image showing a larva after anchor-tipped hairs have been removed (note that the shafts are still present, but the distal portion has been clipped). C) Box plot representing the median, 25–75%, and range of the proportion of trials that larvae remained attached to the textured surface when raised to 90° and 120° with respect to the horizontal plane. Asterisks indicate significant differences (p<0.001).

We gave larvae haircuts to determine if anchor-tipped hairs were necessary for attaching larvae to the nest walls. Larvae that received haircuts were significantly more likely to fall at both 90° and 120° compared to larvae with anchor-tipped hairs intact (Fig. 3; [90°, Mann-Whitney U test; *N* = 10, Z = 3.78, p<0.001], [120°, Mann-Whitney U test; *N* = 10, Z = 3.78, p<0.001]).

Following this test, we conducted a literature search to indentify other species where anchor-tipped hairs were present. Anchor-tipped hairs were only found in species within the ant subfamily Myrmicinae (Table S1), although many species within this subfamily lacked anchor-tipped hairs (Table S2). Anchor-tipped hairs were present in at least 96 species from a total of 22 genera. When the presence or absence of anchor-tipped hairs was mapped onto the myrmicine phylogeny from Moreau & al. [17], we found that anchor-tipped hairs occurred in genera that were widely distributed within the phylogeny. Four genera (*Cephalotes*, *Monomorium*, *Myrmica*, and *Tetramorium*) included both species with anchor-tipped hairs as well as species that lacked anchor-tipped hairs.

Field observations in Gamboa and Barro Colorado Island, Panama revealed that anchor-tipped larval hairs occurred in species that nest in a variety of habitats. *Pheidole* spp. with larvae that had anchor-tipped hairs were found nesting in rotten logs, soil, hollow nuts, and *Tococa* plants with swollen domatia. *Cephalotes* sp. with larvae that had anchor-tipped hairs were also found nesting in *Cecropia* trees.

## Discussion

**Figure 4:**
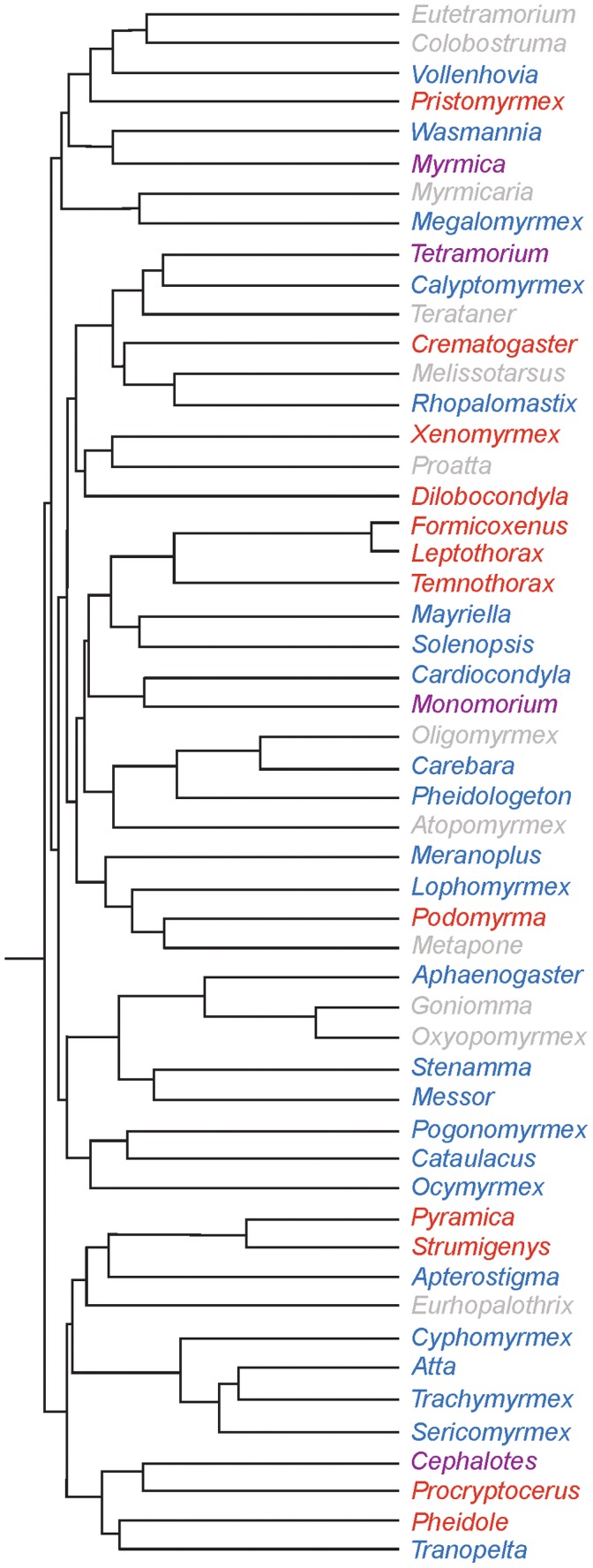
Phylogeny indicating the presence or absence of anchor-tipped hairs in the ant subfamily Myrmicinae. Names listed in red represent genera where larvae possess anchor-tipped hairs, names in blue represent genera without anchor-tipped hairs, names in purple represent genera that include both species with and without anchor-tipped hairs, and names listed in gray represent genera for which we do not have information about anchor-tipped hairs. Phylogeny modified from Moreau et al. [17].

Unlike the arrangement of larvae observed in standard artificial nests, our observations of *P. rhea* revealed that workers hang fourth-instar larvae from the walls and ceiling of their nests when they are provided with chambers that mimic their natural constructions. This behavior was dependent on the presence of specialized anchor-tipped hairs that occur on the dorsal surface of fourth-instar larvae (Fig. 1). When larvae received “haircuts,” they were no longer able to adhere to the nest walls (Fig. 3). Anchor-tipped larval hairs occur in numerous myrmicine genera suggesting that larval hanging behavior is common and widespread in this group (Fig. 4). This behavior and its effects on spatial arrangement could influence how workers feed and care for larvae as well as other worker-larval interactions.

Our observations of larval morphology for *P. rhea* were similar to what has been previously described for *P. bicarinata* [18]. We found evidence of four larval instars with the last instar clearly distinguished by the presence of anchor-tipped hairs (Fig. 2). The presence or absence of anchor-tipped hairs in larvae of *P. rhea* varied with respect to instar and sex. Fourth-instar larvae of both minor workers and soldiers possessed anchor-tipped hairs, while these hairs were absent on early-instar larvae and last-instar males. In contrast to what had been reported for *P. bicarinata*, we found that anchor-tipped hairs in *P. rhea* were arranged in rows of four rather than rows of two [18]. Additionally, male larvae of *P. bicarinata* did have anchor-tipped hairs while males of *P. rhea* did not have anchor-tipped hairs in our observations. We did not include female sexuals in our study, but in *P. bicarinata* female sexual larvae did not have anchor-tipped hairs. This shows that the presence or absence of anchor-tipped hairs is evolutionarily labile, and changes in hair morphology occur across species as well as within species with respect to instar, sex, and caste.

When we placed live colonies of *P. rhea* in nests with textured walls, they hung fourth-instar larvae from the walls of the nest, and this behavior required the presence of intact anchor-tipped hairs. This behavior has been variously reported in other species based on anecdotal observations [12], but this is the first example of direct evidence that anchor-tipped hairs function to suspend larvae from the nest walls. We did observe some cases where early-instar larvae that lack anchor-tipped hairs were attached to the walls of the nest, but this was rare. The cuticle of early-instar larvae has a sticky quality, and this seems sufficient to adhere them to the nest due to their smaller size and mass. In contrast, the largest larvae in the nest–sexuals and soldier larvae–were never observed attached to the nest walls. Soldier larvae possess anchor-tipped hairs, but these hairs were unable to suspend them from the nest walls due to the increased mass of soldier larvae. This may be why anchor-tipped hairs have been lost in males of *P. rhea* and sexual larvae of other *Pheidole* species, where the increased mass of these larvae surpasses the load for which these hairs are capable of supporting.

The presence of anchor-tipped hairs and larval hanging behavior appears to be common and widespread within Myrmicinae, and at least 22 genera display this trait. Anchor-tipped hairs have only been identified within the mymecines, though numerous genera within this group lack these hairs. The genera in which anchor-tipped hairs occur are broadly distributed across the phylogeny (Fig. 4), and it is likely that the development of these hairs reflects an ancestral potential. Of the species where larvae do not possess anchor-tipped hairs, it is possible that specialized rearing conditions may not require a mechanism to hang larvae from the nest walls. One example is the fungus gardening ants, where larvae feed on fungus directly and lack anchor-tipped hairs [19]. In genera where anchor-tipped hairs occur, however, the question remains: what is the adaptive significance of hanging larvae inside the nest?

One possibility could be that specific environmental conditions, whether they be related to climate or nesting habitat, may specify a benefit to hanging larvae from the nest walls. A comparison of life-history traits among species that possess anchor-tipped hairs, however, did not support this. With respect to climate, *P. rhea* is found in a desert environment, but anchor-tipped hairs are present in species that inhabit a wide diversity of habitats, including temperate forests (e.g. *Myrmica brevispinosa*, *Temnothorax nevadensis*) and tropical regions (e.g. *Cephalotes atratus*, *Strumigenys epinotalis*). *P. rhea* is ground nesting, but there are other species that possess anchor-tipped hairs that nest in decaying logs, rock cavities (e.g. *Temnothorax spp.*), or are specialized arboreal nesters (e.g. *Cephalotes spp.*). Despite the lack of correlation between the presence of anchor-tipped hairs and a single nesting habitat, there is at least some evidence that the ability to hang larvae from the nest walls may be particularly important to species that nest arboreally. Of the myrmicine genera that specialize on arboreal habitats (e.g. *Cephalotes*, *Xenomyrmex*, and *Crematogaster*), most possess anchor-tipped hairs. Further evidence comes from species outside of the myrmicines, where anchor-tipped hairs do not occur. Many of these arboreal nesting species possess single-hooked hairs that could serve a similar function as anchor-tipped hairs. For example, larvae of the arboreally nesting pseudomyrmecines possess extended “single-hooked” (uncinate) hairs that are distributed on their dorsal surface similar to the distribution of anchor-tipped hairs in myrmicine genera [12]. Similarly, the dolichoderine species *Azteca alfari*, which lives inside living trunks of *Cecropia*, has single-hooked hairs that may be used to hang larvae from the interior walls of their host plant [12]. Species that have the ability to hang larvae from the walls of their host plants would be able to hang larvae inside branches that are slanted or even vertical whereas larvae lacking these hairs may not be able to rest on these surfaces.

In addition to anchor-tipped and single-hooked hairs there may be other larval hair morphologies that allow larvae to hang from the nest walls. The “sticky doorknobs” present on the larvae of *Hypoponera* have been described as a feature used to hang larvae from the walls of their underground nests [20], [21], and various other ponerine species possess single-hooked hairs [12]. With respect to *Hypononera*, Rüger et al. [22] hypothesized that workers hang larvae on the walls of their nest to prevent them from cannibalizing neighboring larvae. These authors hypothesized that larvae may attempt to selfishly develop into queens by gaining excess resources, and hanging larvae on the nest walls may prevent larvae from cannibalizing their neighbors. Conflict over caste determination may exist in species such as *Hypoponera* where larvae remain bipotential, but queen determination in *Pheidole* occurs inside the egg [23]. While it is not clear what factors initially selected for anchor-tipped hairs in the myrmicines, it is unlikely that the current function of these hairs relates to caste conflict.

Evidence of convergent evolution for hair morphologies associated with larval hanging behavior as well as the widespread distribution of anchor-tipped hairs among myrmicine genera suggests that hanging larvae from the nest walls serves a general purpose. Many ant species meticulously transport brood to track optimal temperature and humidity conditions [24], [25], [26], and it is possible that hanging larvae on the ceiling of chambers could serve some thermoregulatory purpose. In both *P. rhea* and *P. bicarinata*, anchor-tipped hairs are only associated with fourth-instar larvae, which represent the primary growth stage during larval development. Hanging larvae in a single layer on the nest walls may allow workers to position them so that they receive optimal temperature conditions or provide better access to nurse workers for feeding larvae. One reason suggested for why medium sized larvae of *Temnothorax* were grouped on the outside of the larval pile was that this position could provide nurse workers with better access to these larvae for feeding [9]. Because early-instar larvae may not require the same access to food as fourth-instar larvae, this may be one reason why anchor-tipped hairs are absent in early-instars. Incidentally, *Temnothorax* is a genus where larvae possess anchor-tipped hairs, so grouping medium-sized larvae on the outside of the brood pile may be related to their tendency to hang larvae in their natural nests. Studying *Temnothorax* in nests with textured walls may reveal new information about how these ants organize their brood under natural conditions and could provide further insight into their brood-sorting behavior. Hypotheses about the adaptive nature of hanging larvae from the nest walls remain untested, but future examinations of larval organization may shed light on the function of this behavior.

While larvae appear to be complicit recipients of adult care, they are also known to influence the expression of adult behaviors such as task performance [27], [28], nest construction [29], queen egg-laying rate, and colony growth [30]. Spatial organization of larvae in the nest may affect how larvae and workers interact and could influence colony-level traits. The way larvae are grouped in colonies of the bumble bee *Bombus impatiens* determines how larvae are fed: larvae at the periphery of the nest are fed less frequently by workers than larvae reared in cells located centrally [31]. An indirect result of this spatial arrangement is the production of a polymorphic worker caste, which ultimately leads to differences in adult task specialization based on worker size [32]. Understanding how the spatial organization inside the nest contributes to colony-level traits is important to understanding social regulation of collective behavior. Observing ants in nests that allow them to express natural behaviors, such as hanging larvae on the nest walls, should provide new insight into social behavior and be taken into account in future studies of social insect behavior.

## Supporting Information

Table S1 **Species in Myrmicinae that possess anchor-tipped hairs.**
(DOCX)

Table S2 **Species in Myrmicinae that lack anchor-tipped hairs.**
(DOCX)
