# Supplementary material for: The Role of Anchor-Tipped Larval Hairs in the Organization of Ant Colonies
Source: PLoS One. 2012 Jul 25;7(7):e41595. doi: 10.1371/journal.pone.0041595 (PMC3404993; doi:10.1371/journal.pone.0041595)
Supplement: Table S1 — Species in Myrmicinae that possess anchor-tipped hairs. (DOCX) [file pone.0041595.s001.docx]

**Table S1.** **Species in Myrmicinae that possess anchor-tipped hairs^*†^**

| **Genus** | **Species** |
| --- | --- |
| ***Acanthognathus*** [1] | *A. rudis* |
| ***Anergates*** [2] | *A. atratulus* |
| ***Cephalotes*** [3] | *C. atratus*, *C. basalis*, *C. clypeatus*, *C. maculatus*, *C. minutus*, *C. pusillus*, *C. umbraculatus*, *C. varians*, *C. wheeleri* |
| ***Crematogaster*** [4,5] | *C. acuta* [4], *C. auberti* [4], *C. australis* [5], *C. crinosa* [4], *C. depilis* [5], *C. difformis* [4], *C. laeviuscula* [4], *C. limata* [4], *C. lineolata* [4], *C. menilekii* [4], *C. victima* [5] |
| ***Dilobocondyla*** [6] | *D. chapmani* |
| ***Formicoxenus*** [7,8] | *F. nitidulus* [7], *F. provancheri* [8] |
| ***Harpagoxenus*** [7] | *H. sublaevis* |
| ***Leptothorax*** [7] | *L. acervorum*, *L. muscorum* |
| ***Liomyrmex*** [2] | *L. getroi* |
| ***Monomorium*** [2] | *M. antarcticum* |
| ***Myrmica*** [9] | *M. americana*, *M. brevispinosa*, *M. dshungarica*, *M. emeryana*, *M. incompleta*, *M. lobicornis*, *M. monticola*, *M. rubra*, *M. sabuleti*, *M. schenki* |
| ***Nesomyrmex*** [7] | *N. echinatinodis* |
| ***Pheidole*** [2,10,11,12] | *P. bicarinata* [10], *P. brevicornis* [11], *P. californica* [11], *P. longipes* [12]*, P. megacephala* [12]*, P. micula* [11]*, P. moerens* [11]*, P. neokohli* [2]*, P. noda* [12]*, P. pilifera* [12]*, P. punctulata* [12]*, P. rhea, P. tepicana* [12] |
| ***Podomyrma*** [6] | *P. adelaidae* |
| ***Pristomyrmex*** [5,6] | *P. punctatus* [6], *P. quadridentatus* [5] |
| ***Procryptocerus*** [3,8] | *P. adlerzi* [8], *P. pictipes* [3], *P. regularis* [8], *P. schmalzi* [3] |
| ***Protomognathus*** [7] | *P. americanus* |
| ***Rogeria*** [8] | *R. procera* |
| ***Strumigenys*** [13] | *S. elongata*, *S. epinotalis*, *S. lewisi*, *S. louisianae*, *S. nidifex*, *S. nigrescens*,  *S. pergandei*, *S. perplexa*, *S. rostrata*, *S. schulzi*, *S. szalayi, S. talpa* |
| ***Temnothorax*** [7,8] | *T. ambiguus* [7], *T. bermudezi* [7], *T. carinatus* [8], *T. congruus* [7], *T. exilis* [7], *T. hispidus* [8], *T. nevadensis* [8], *T. nitens* [8], *T. obturator* [7], *T. rugatulus* [7], *T. semiruber* [7], *T. unifasciatus* [7], *T. wheeleri* [7] |
| ***Tetramorium*** [7,14] | *T. aculeatum* [7], *T. caespitum* [14], *T. guineense* [14], *T. punicum* [14], *T. schmidti* [14], *T. striativentre* [14], *T. turneri* [14] |
| ***Xenomyrmex*** [15] | *X. stollii* |

*Uptdated species names based on www.antbase.org, 2012

^†^Numbers indicate references
