# Supplementary material for: The Role of Anchor-Tipped Larval Hairs in the Organization of Ant Colonies
Source: PLoS One. 2012 Jul 25;7(7):e41595. doi: 10.1371/journal.pone.0041595 (PMC3404993; doi:10.1371/journal.pone.0041595)
Supplement: Table S2 — Species in Myrmicinae that lack anchor-tipped hairs. (DOCX) [file pone.0041595.s002.docx]

**Table S2.** **Species in Myrmicinae that lack anchor-tipped hairs^*†^**

| **Genus** | **Species** |
| --- | --- |
| ***Acromyrmex*** [16] | *A. hystrixi*, *A. lundii*, *A. octospinosus* |
| ***Adlerzia*** [11] | *A. froggatti* |
| ***Allomerus*** [2] | *A. octoarticulatus* |
| ***Aphaenogaster*** [11,12] | *A.* (*Novomessor*) *albisetosa* [12], *A.* (*Novomessor*) *cockerelli* [11], *A. flemingi* [11], *A. inermita* [12], *A. megommata* [11], *A. pythia* [11], *A. rudis* [12], *A. subterranea* [12], *A. tennesseensis* [12], *A. texana* [12] |
| ***Apterostigma*** [16] | *A. collare*, *A. mayri* |
| ***Atta*** [16] | *A. cephalotes*, *A. colombica*, *A. sexdens*, *A. texana* |
| ***Basiceros*** [13] | *B. grave*, *B. militaris* |
| ***Calyptomyrmex*** [5] | *C. piripilis* |
| ***Cardiocondyla*** [17] | *C. ulianini* |
| ***Carebara*** [18] | *C. anophthalma*, *C. corniger*, *C. jacobsoni*, *C. mjobergi*, *C. sundaica*, *C. termitolestes*, *C. thoracica* |
| ***Cataulacus*** [3] | *C. egenus*, *C. horridus*, *C. taprobanae* |
| ***Cephalotes*** [8] | *C. pallens* |
| ***Cyphomyrmex*** [16] | *C. rimosus*, *C. strigatus* |
| ***Daceton*** [13] | *D. armigerum* |
| ***Epopostruma*** [13] | *E. alinodis*, *E. laevigata* |
| ***Lophomyrmex*** [18] | *L. quadrispinosus* |
| ***Manica*** [11] | *M. bradleyi*, *M. hunteri*, *M. invidia*, *M. rubida* |
| ***Mayriella*** [5] | *M. hackeri* |
| ***Megalomyrmex*** [2] | *M. symmetochus* |
| ***Meranoplus*** [5,14] | *M. dimidiatus* [5], *M. oceanicus* [14] |
| ***Messor*** [11,12,19] | *M.* (*Veromessor*) *andrei* [11], *M. barbarus* [12], *M.* (*Veromessor*) *chamberlini* [11]*, M.* (*Veromessor*) *lobognathus* [19], *M.* (*Veromessor*) *pergandei* [11]*, M.* (*Veromessor*) *smithi* [11]*, M. striaticeps* [12] |
| ***Monomorium*** [2,5] | *M. afrum* [2], *M. chilensis* [5], *M. floricola* [2], *M. laeve* [5], *M. pharaonis* [2] |
| ***Myrmecina*** [5,6] | *M. americana* [6], *M. australis* [5], *M. graminicola* [6] |
| ***Myrmica*** [20] | *M. colax* |
| ***Myrmicocrypta*** [16] | *M. spinosa*, *M. urichi* |
| ***Ocymyrmex*** [8] | *O. fortior* |
| ***Orectognathus*** [13] | *O. clarki*, *O. mjobergi*, *O. satan*, *O. versicolor* |
| ***Oxyepoecus*** [5] | *Oxyepoecus sp.* |
| ***Pheidologeton*** [18] | *P. diversus* |
| ***Pogonomrymex*** [9,11] | *P.* (*Ephebomyrmex*) *huachucanus* [11], *P.* (*Ephebomyrmex*) *imberbiculus* [9]*, P.* (*Ephebomyrmex*) *naegelii* [11]*, P. barbatus* [9]*, P. mayri* [9]*, P. occidentalis* [9]*, P. salinus* [11] |
| ***Recurvidris*** [18] | *R. kemneri* |
| ***Rhopalomastix*** [17] | *R. rothneyi* |
| ***Sericomyrmex*** [16] | *S. amabilis* |
| ***Solenopsis*** [2] | *S.* (*Diplorhoptrum*) *fugax*, *S.* (*Diplorhoptrum*) *molesta*, *S.* (*Diplorhoptrum*) *texanum*, *S. geminata*, *S. picea*, *S. tenuis*, *S. xyloni* |
| ***Stenamma*** [11,12] | *S. diecki* [12], *S. manni* [11], *S. westwoodii* [12] |
| ***Tetramorium*** [8] | *T. lanuginosum* |
| ***Trachymyrmex*** [16] | *T. opulentus*, *T. septentionalis* |
| ***Tranopelta*** [2] | *T. gilva* |
| ***Vollenhovia*** [2] | *V. oblonga pedestris* |
| ***Wasmannia*** [14] | *W. auropunctata* |

*Uptdated species names based on www.antbase.org, 2012

^†^Numbers indicate references
